# Does propolis affect the quality of life and complications in subjects with irritable bowel syndrome (diagnosed with Rome IV criteria)? A study protocol of the randomized, double-blinded, placebo-controlled clinical trial

**DOI:** 10.1186/s13063-020-04615-5

**Published:** 2020-08-05

**Authors:** Mahsa Miryan, Pezhman Alavinejad, Mohammadreza Abbaspour, Davood Soleimani, Alireza Ostadrahimi

**Affiliations:** 1 Nutrition Research Center, Student Research Committee, Department of Clinical Nutrition, School of Nutrition and Food Sciences, Tabriz University of Medical Sciences, Tabriz, Iran; 2 Nutrition Research Center, Department of Clinical Nutrition, School of Nutrition and Food Sciences, Tabriz University of Medical Sciences, Tabriz, Iran; 3 Alimentary Tract Research Center, Ahvaz Imam Hospital, Ahvaz Jundishapur University of Medical Sciences, Ahvaz, Iran; 4 Targeted Drug Delivery Research Center, Pharmaceutical Technology Institute, Mashhad University of Medical Sciences, Mashhad, Iran; 5 Nutritional Sciences Department, School of Nutrition Sciences and Food Technology, Kermanshah University of Medical Sciences, Kermanshah, Iran

**Keywords:** Gastrointestinal diseases, Irritable bowel syndrome, Propolis, Anti-inflammatory agent

## Abstract

**Background:**

Irritable bowel syndrome (IBS) is one of the most frequent and recurrent gastrointestinal diseases. However, up to now, no pharmacological agent has been approved to treat IBS. Emerging evidence showed that inflammation has a vital role in enhancing nervous system sensitivity and perception of abdominal pain in subjects with IBS. Propolis is an herbal substance with a broad spectrum of antioxidants, anti-inflammatory, and prebiotic properties, which might exert beneficial effects to reduce the severity of IBS. The current clinical trial aims to evaluate the efficacy of propolis supplementation on IBS.

**Methods:**

This single-center, randomized, double-blind, placebo-controlled clinical trial will be performed to evaluate the effect of propolis supplementation in adult patients with IBS diagnosed with Rome IV criteria. Fifty-two eligible patients will randomly be allocated to receive a propolis tablet (450 mg, containing 100 mg polyphenol compounds) or identical placebo, twice daily for 6 weeks. The primary outcome of the trial is an improvement in IBS severity from baseline to the sixth week of intervention. The secondary outcomes include the change in weight, waist circumference, and IBS quality of life. We will use the paired sample *t* test or Mann-Whitney *U* test for the within-group comparison and independent sample *t* test or Wilcoxon rank-sum and chi-square test or Fisher’s exact test for the between-group comparison. Besides, a multivariable-adjusted mean effect will be computed using the ANCOVA test.

**Discussion:**

We hypothesize that propolis supplementation would be useful for treating IBS through its antioxidants, anti-inflammatory, and prebiotic properties. This trial will show the results of propolis supplementation, whether positive or negative, on IBS. If the current trial confirms our hypothesis, propolis supplementation can be a new choice in adjunctive therapy of IBS.

**Trial registration:**

Iranian Registry of Clinical Trials IRCT20190708044154N1. Registered on 26 December 2019. Updated on 13 February 2020. https://en.irct.ir/trial/40983

**Sponsor:**

Tabriz University of Medical Sciences, Tabriz, Iran

## Background

Irritable bowel syndrome (IBS) is one of the most common and recurrent gastrointestinal (GI) diseases with unexplained abdominal discomfort and bowel habit changes [1]. There has been no documentation of structural bowel dysfunction or metabolic disorders in patients with IBS, and diagnosis is made based on the Rome IV criteria as the latest and newest diagnostic tool for IBS [2, 3]. According to the Bristol Stool Form Scale (BSFS), IBS is classified into diarrhea-predominant (IBS-D), constipation-predominant (IBS-C), and mixed form (IBS-M). This functional bowel disease affects approximately 10% of the general population [4].

To date, the exact pathogenic mechanisms of IBS have not been completely clarified. However, several factors are expected to play a role in IBS etiology, including microscopic inflammation, enhancement of GI tract permeability, alterations of the nervous system (such as abnormal GI motility and visceral hypersensitivity), chronic infections, genetics, psychosocial stress, changes in brain-gut axis, and also alterations of gut flora [5–8]. Besides, oxidative stress and inflammation have a role in the augmentation of abdominal pain perception and nervous system sensitivity in these patients [8]. Various therapeutic approaches to IBS treatment have been suggested due to possible pathogenic mechanisms. However, up to now, no pharmacological agent has been approved to treat all IBS symptoms or all IBS forms [9]. Recent clinical evidence indicates that prebiotics and probiotics might improve the IBS symptoms severity through modulating the mucosal immune response [10, 11]. Furthermore, some plant metabolites, such as polyphenol compounds, have been shown to maintain GI function and the body’s health due to gut-microbiota modulation and their anti-inflammatory and antioxidant properties [12, 13].

Propolis, a natural resin, is produced by bees from petals and plant buds. It is a complex substance mainly made up of polyphenol compounds and terpenes, with a broad spectrum of antioxidants, anti-inflammatory, and prebiotic properties [14]. Recent evidence indicates that the administration of propolis reduces the histopathological scores of colon damage, increases the mucin levels, and suppresses colonic inflammation in colitis animals [15–17]. In rats with obstructive jaundice, propolis exerts protective effects against intestinal villus atrophy and bacterial translocation [18]. Moreover, some studies showed that propolis increases the secretion of mucin and mucosal prostaglandin E_2_ levels [19], modulates gut microbiota, and reduces the levels of circulating endotoxin and expression of toll-like receptor gene in animals [20]. Further efforts in this area also showed that propolis could improve the intestinal barrier function through the upregulation of the intestinal tight junction proteins gene [21].

### Study rationale

Considering to the tight relationship between inflammation, oxidative stress, dysbiosis of the gut microbiota, and alteration in the intestinal permeability with IBS, it seems that propolis supplementation would contribute to improve the health of the gastrointestinal system in patients with IBS. Most clinical trials reported the safety and efficacy of propolis supplementation on glycemic control in patients with type 2 diabetes mellitus [22]. To the best of our knowledge, there is no clinical trial investigating the effect of propolis in patients with IBS.

### Objectives

The current clinical trial aims to evaluate the effect of propolis supplementation on the IBS Severity Index (IBSSI). Also, secondary objectives of this trial include the changes in weight, body mass index (BMI), waist circumference (WC), IBS quality of life (IBS-QOL), and anxiety with propolis supplementation compared to placebo. For controlling of confounder factors, changes in dietary intakes, physical activity levels, and anxiety will be assessed in the current trial.

## Materials and methods

### Study design

The current study is a prospective, single-center, randomized, double-blind, placebo-controlled clinical trial evaluating the efficacy of propolis supplementation in subjects with IBS. This clinical trial will be performed at the gastrointestinal clinic, a referral center for gastrointestinal diseases in Ahvaz, the southwest of Iran. All volunteers will provide written informed consent before participation in this randomized clinical trial.

**Fig. 1:**
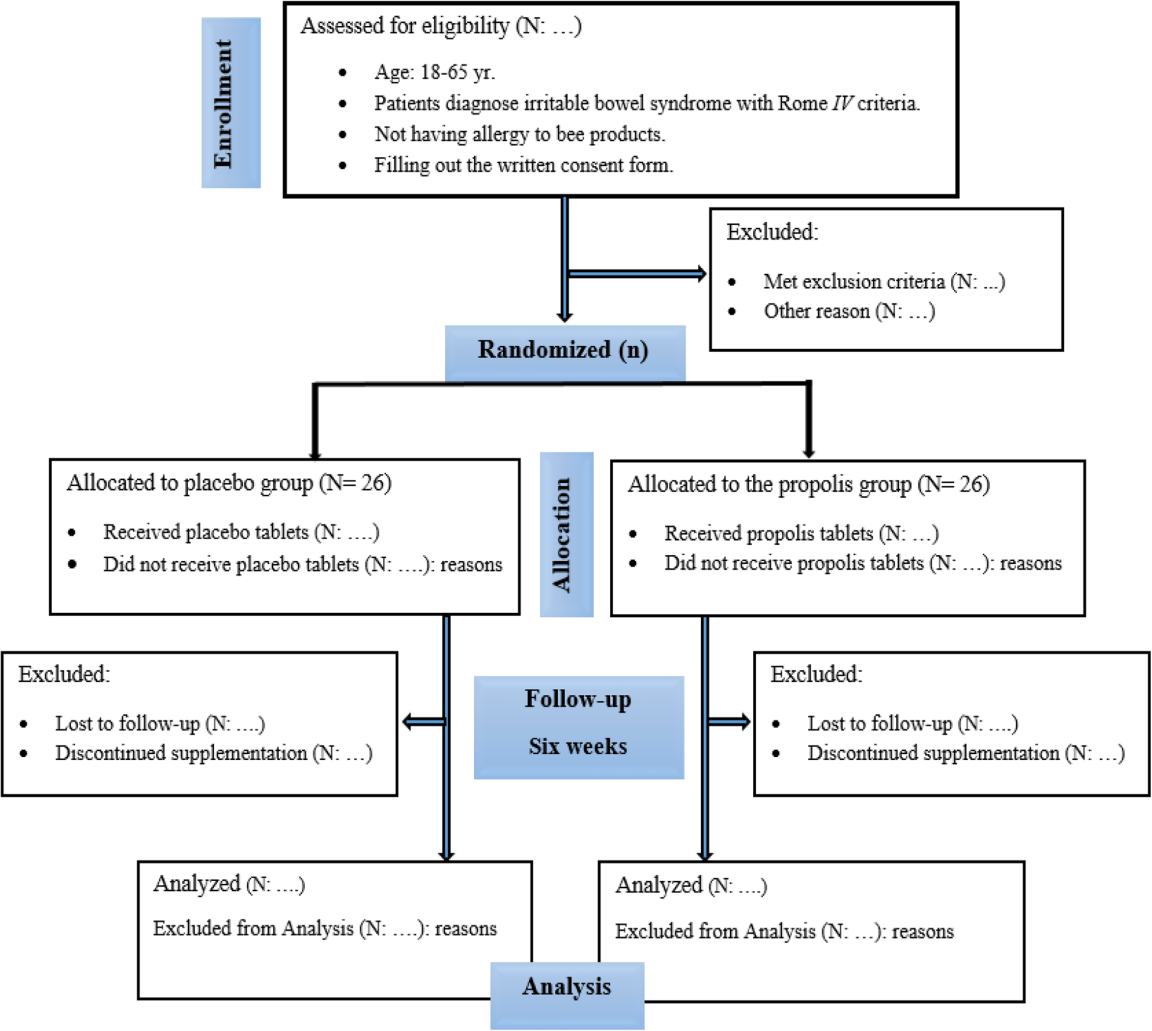
Study flow chart of enrolment, allocation, intervention, and assessment

The current trial is registered at the Iranian Registry of Clinical (ID: IRCT20190708044154N1). The study protocol was drafted and developed in accordance with the Standard Protocol Items: Recommendations for Interventional Trials (SPIRIT) 2013 checklist (Additional file 1). The protocol flow chart and timeline of the study are shown in Fig. 1 and Table 1, respectively. A protocol deviation is accidental and unintentional changes to the study protocol which have no significant impact on the study’s risk or benefit, subject’s rights, safety or welfare, and the integrity of the data [23]. The protocol deviations may result from the action of the patients or researchers. Examples of the protocol deviation for this study include:

- A rescheduled study visit
- Failure to collect an ancillary self-report questionnaire
- Subject’s refusal to complete scheduled research activities.

**Table 1:** Timeline of the trial

Explanation of the trial activities	Time (month)											
	1	2	3	4	5	6	7	8	9	10	11	12
Material preparation	*	*	*	*	*							
Recruitment						*	*					
Intervention								*				
Data analysis									*	*		
Writing the final report of the trial											*	*
The expected time	*	*	*	*	*	*	*	*	*	*	*	*

Any amendments of this study protocol which have related to safety and benefit of participants will need to be agreed upon by Department of Clinical Nutrition in Tabriz University of Medical Sciences and approved by the Medical Ethics Committee of Tabriz University of Medical Sciences, Tabriz, Iran, before the implementation of the study. Any change in the study protocol will be sent to Trial Journal (www.trialsjournal.biomedcentral.com).

### Study setting

This clinical trial will be performed at the gastrointestinal clinic, a referral center for gastrointestinal diseases in Ahvaz, the southwest of Iran. All participants will be informed of this study through advertisements on the clinic’s television.

### Study participants and recruitment

Volunteers will be recruited from outpatients referred to the gastrointestinal clinic in Ahvaz, Iran, through advertisement on the clinic’s television. Patients who meet the international *Rome IV* criteria for diagnosing IBS will be assessed for eligibility criteria, after differentiating IBS from structural bowel disorders by an expert gastroenterologist (Dr. PM) at this clinic. The international *Rome IV diagnostic Questionnaire* is the well-known and latest diagnostic instrument in the field of IBS in clinical researches. According to *Rome IV* criteria, individual who had recurrent abdominal pain or discomfort at least 1 day/week in the last 3 months with symptom onset at least 6 months before a diagnosis has IBS if she/he had at least two of the following criteria: symptom improvement with defecation, onset simultaneous with a change in the frequency, or/and onset simultaneous with a change in the form or appearance [3].

### Inclusion criteria

The inclusion criteria will consist of patients who have an age between 18 and 65 years, who have an IBS-C or IBS-M (much more common forms of IBS) according to the BSFS, and who fill out a written informed consent to participation in this trial.

### Exclusion criteria

The exclusion criteria will consist of patients who are a pregnant or breastfeeding, who have/had a malignancy or other chronic digestive diseases such as IBD, who have regular use of drugs that modify GI movements (such as metoclopramide, cisapride, narcotics, and diphenoxylate), who have regular use of laxatives or antibiotics, who have regular use of the prebiotic or probiotic supplement, who have regular use of antidepressant drugs, who have a history of major surgery in the gastrointestinal tract (Billroth’s operation, having an ostomy and any resection of any part of the digestive tract), who underwent psychotherapy, who have a restricted diet (such as vegetarian diets), and who have/had an allergy to bee products.

Participants who are unwilling to continue collaborating on the study, have the sensitivity to propolis supplement, become pregnant, alter the patient’s disease to other gastrointestinal diseases, or have poor compliance with the assigned treatment (less than 80%) will be withdrawn from follow-up.

### Randomization and blinding

Stratified permuted block randomization will be used to balance gender and IBS types between two groups over time. Four strata is constructed according to sex and IBs types as follows:

- Stratum1: Male patients with IBS-C
- Stratum 2: Female patients with IBS-C
- Stratum 3: Male patients with IBS-M
- Stratum 4: Female patients with IBS-M

Within each stratum mentioned above, eligible patients will be randomly allocated in a ratio of 1:1 to receive propolis or placebo with a block size of 4. A block size of 4 is constructed six different arrangements (block) to assign patients to the trial groups as follows equally:

- Block: Propolis, Propolis, Placebo, Placebo
- Block: Placebo, Placebo, Propolis, Propolis
- Block: Placebo, Propolis, Placebo, Propolis
- Block: Propolis, Placebo, Propolis, Placebo
- Block: Propolis, Placebo, Placebo, Propolis
- Block: Placebo, Propolis, Propolis, Placebo

The sequence of the blocks mentioned above will be prepared for each stratum using a random numbers table. The study pharmacist will provide the placebo tablet similar to propolis tablet in color, odor, taste, shape, size, and weight, and they will be held in drug containers. All containers will be the same in terms of shape, color, odor, size, and weight and will be kept inside consecutively numbered, opaque, sealed envelopes, which are completely impermeable to light.

The trial-group assignments will be concealed from patients, physician, and investigators (enrolling, assessing, and analyzing) until the end of the study and data analysis, with the exception of the study pharmacist. The study pharmacist will provide a randomization list, sequentially numbered drug containers, and opaque and sealed envelopes.

### Study interventions

Fifty-two eligible patients will be randomly assigned to either the intervention group (*n* = 26) or the control group (*n* = 26). Patients in the intervention group will receive an identical propolis tablet containing 450 mg Iranian green propolis extract (100 mg polyphenol compounds and 67 mg flavonoids) twice a day, one tablet before lunch and one tablet before dinner, for 6 weeks. Propolis sample is obtained from honey bee colonies located in Rasht, the north area of Iran. Patients in the control group will receive an identical placebo tablet twice a day, before lunch and dinner, for 6 weeks. Propolis and placebo tablets will be similar in color, odor, taste, shape, size, and weight and will be produced by the School of Pharmacy, Mashhad University of Medical Sciences, Iran. Patients will be asked to continue their usual diet, physical activity, and medicines until intervention completion. Also, they will be instructed on how to use their supplements. Patients will be followed through weekly phone calls and intermediate visits (t_3_) to the clinic for assessing treatment compliance and adverse events. At the end of the trial (t_7_), participants will be informed of the usual dietary recommendations of IBS.

### Outcomes

The primary outcome of this clinical trial is the change in the severity of IBS from baseline to the sixth week of the intervention. The secondary outcomes of this clinical trial are the change in IBS quality of life, weight, and waist circumference from baseline to the sixth week of the intervention.

Demographic variables, including sex, age, race, education, income, marital status, smoking status, housing status, employment status, medical history, and family history, will be obtained from each participant at the beginning of the study (t_0_). Besides, for controlling of confounder factors, changes in dietary intakes, physical activity level, and anxiety will be evaluated in the current trial.

At the beginning of the trial, demographic information, food intakes, physical activity, weight, height, BMI, waist circumference, anxiety, IBS quality of life, and IBS severity will be collected by validated tools.

### Instruments

#### IBSSI

The study gastroenterologist who will be blinded to the treatment assignment will assess the IBS severity with the use of the IBS Severity Index (IBSSI). This scale is designed by Francis et al. [24]. This is a validated questionnaire with five sub-scales that measures the intensity and frequency (number of days) of abdominal pain, the intensity of abdominal distension, bowel movements satisfaction, and potential impact of IBS on the patient’s daily life during the last 10 days. The mean score of each section is a maximum of 100, and the total score of the questionnaire is a maximum of 500. Mild, moderate, and severe cases are displayed with scores of 75 to 175, 175 to 300, and more than 300, respectively. The internal correlation coefficient of 0.86 and Cronbach’s alpha of 0.69 have been reported for this questionnaire [25]. Although none of the IBS symptom severity tools have been completely validated, the above tool is currently reported to be the best tool used in most similar studies.

#### IBS-QOL

The study gastroenterologist who will be blinded to the treatment assignment will assess patients’ quality of life with the use of the IBS quality of life (IBS-QOL) questionnaire. This is a validated questionnaire with 34 questions and eight sub-scales including blame, interference with daily activities, physical imagery, health concerns, eating avoidance, social reactions, sexual problems, and communication issues that are in a range of degrees (never, rarely, to some extent, high, and severe). The minimum and maximum scores are 34 and 170; respectively. According to this questionnaire, lower scores indicate a higher quality of life. Daryani et al. validated this questionnaire. Its content validity has been confirmed by several medical groups, psychiatry, psychology, and epidemiology in Iran. The validity of the questionnaire was also evaluated using Cronbach’s alpha coefficient (*α* = 0.92), and the internal consistency coefficient of the questionnaire was excellent (*α* = 0.96). Also, its correlation coefficient with severity of symptoms was significant (*r* = 0.67; *P* value < 0.01) [26].

#### Anthropometrics

**Fig. 2:**
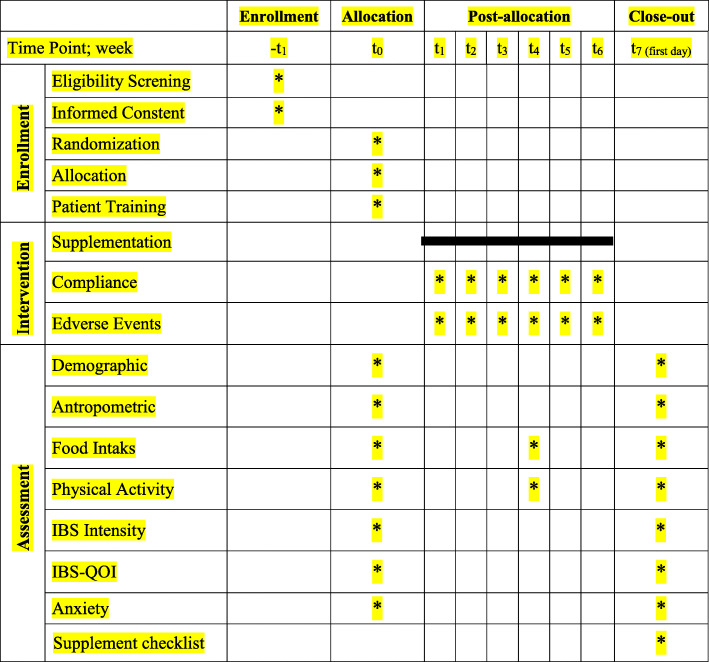
Schedule of enrollment, intervention, and assessments based on SPIRIT guidelines. Abbreviation: SPIRIT, Standard Protocol Items: Recommendations for Interventional Trials; IBS, irritable bowel syndrome; IBS-QOI, IBS quality of life; IBSSI, IBS Severity Index. Demographic variables, including sex, age, race, education, income, marital status, smoking status, housing status, employment status, medical history, and family history

A trained nutritionist who will be blinded to the treatment assignment will assess anthropometric variables, including weight, height, body mass index (BMI), and waist circumference (WC) (Fig. 2). Weight will be measured using a digital Seca scale (Saca 831, Hamburg, Germany), with light clothing, to the nearest 100 g. Height will be measured using a portable stadiometer (Seca, Hamburg, Germany) in the standing position to the nearest 0.5 cm. Then, BMI will be calculated by dividing weight in kilograms by height in meters squared. WC will be measured without clothing at a level midway between the lower rib margin and the iliac crest at the end of exhalation and standing position using tape, to the nearest 0.5 cm.

#### Three-day food records

The change in dietary intakes will be measured using a 3-day food record (2 weekdays and 1 weekend day), as the “gold standard” for dietary assessment [27], at the beginning (t_0_), the middle (t_3_), and the end of the intervention (Fig. 2). A trained nutritionist who will be blinded to the treatment assignment will instruct patients on how to complete their food records and then will analyze completed food records. The amounts of each food item and drink will be converted to gram/day using Iranian Household Measures and then will be converted to the value of nutrients, such as FODMAPs (fermentable oligosaccharides, disaccharides, monosaccharides, and polyols), and calorie using Nutritionist IV software (First Databank Inc., Hearst Corp., San Bruno, CA, USA).

#### IPAQ

The change in physical activity will be measured using the international physical activity questionnaire (IPAQ) at the beginning and the end of the intervention. A trained nutritionist who will be blinded to the treatment assignment will instruct patients on how to complete their IPAQ and then will analysis completed IPAQ according to its guideline. IPAQ is an instrument designed for population surveillance of physical activity among adults which consists of seven questions about all types of physical activity as part of daily life performed in the last 7 days [28]. The Farsi-translated version of IPAQ was validated among the Iranian population by Moghaddam et al. with an excellent internal consistency (Cronbach’s alpha of 0.9). The reliability is reported by the Spearman-Brown correlation of 0.3 [29]. According to the scoring guideline of this questionnaire, the physical activity of the participants will be expressed as metabolic equivalents (MET)-minutes/week and four levels of activity, including vigorous, intermediate, walking, and sitting [30].

#### BAI

The change in anxiety will be measured using the Beck Anxiety Inventory (BAI) at the beginning and the end of the intervention (Fig. 2). A trained psychologist who will be blinded to the treatment assignment will instruct patients on how to complete their BAI questionnaire. This questionnaire is designed to measure anxiety with 21 items. Each item expresses one of the symptoms of anxiety that is commonly experienced by patients who are clinically anxious or those who are in anxious conditions [31]. The internal consistency coefficient of the questionnaire was excellent (Cronbach’s alpha of 0.92) [32]. The validity of the questionnaire showed good validity of the questionnaire by comparing the quantitative evaluation of clinical specialist with the scores of the subjects’ performance (*r* = 0.72; *P* value < 0.001) [33].

### Sample size calculation

We used Hern’s single-stage phase II methodology to calculate the sample size [34]. Based on results from other dietary supplements in patients with IBS [35] in which 15% of patients in the placebo group (*P*_0_) and 40% of patients in the intervention group (*P*_*1*_) had complete remission from symptoms of IBS, we need 22 patients in each trial group with a power of 80%. To compensate for withdrawal during follow-up, we increase the final sample size by 20%, to 26 patients in each group.

### Safety evaluation

In this study, the dose of propolis supplementation was extrapolated from the study conducted by Sabuncuoglu et al. [18] in which the administration of propolis at a dose of 100 mg kg^−1^ improves the function of the GI tract and gut microbiota in rats. Based on the following formula for the conversion of animal dose to human [36] and reference body weight of 60 kg, the dose of propolis for a human is approximately 900 mg. At this dose of propolis, no adverse events were reported in the previous clinical trials.

$$ {\displaystyle \begin{array}{c}\mathrm{Human}\ \mathrm{equivalent}\ \mathrm{dose}\ \left(\mathrm{mg}/\mathrm{kg}\right)=\mathrm{Animal}\ \mathrm{dose}\left(\mathrm{mg}/\mathrm{kg}\right)\times \left({\mathrm{km}}_{\mathrm{Animal}}/{\mathrm{km}}_{\mathrm{Human}}\right)\\ {}\mathrm{Animal}\ \mathrm{dose}=100\ \mathrm{mg}/\mathrm{kg}\kern0.5em {\mathrm{km}}_{\mathrm{rat}}=6\kern0.5em {\mathrm{km}}_{\mathrm{Human}}=37\end{array}} $$

Patients will be asked to avoid taking the assigned supplement following the occurrence of adverse events and to immediately call the study physician or other investigators. One of the study researchers will be instructed to call the participants weekly by phone and ask them about experiencing any adverse events. Tabriz University of Medical Sciences is responsible to follow up on any adverse events and reports them to the Medical Ethics Committee of Tabriz University of Medical Sciences for decision making. If the reported adverse events are related to the consumed supplements, the study will be stopped and patients will be referred to a specialist for immediate treatment in which can be treated free of charge. Participants who are unwilling to continue collaborating on the study will be able to discontinue the trial at any time.

### Data management

One of the researchers will check the coding, security, and storage of data. Also, she/he will check data entry, and data values double times. If any patient reports the occurrence of adverse events, more information is needed for the decision making about excluding the patient from the study.According to the Medical Ethics Committee Criteria, unblinding is permissible in this situation, and we can specify whether the patient has received the propolis or placebo.

### Adherence to the intervention

All patients will be instructed on how to use their supplements at the baseline, each phone call, and intermediate visits (t_3_). All patients will be asked to complete a daily calendar immediately after consumption of their assigned treatments. The adherence to intervention refers to the degree to which the behavior of trial participants corresponds to the intervention assigned to them. Adherence to the assigned treatments will be checked by means of a daily calendar at each phone call and will be rechecked by counting the returned tablets at the end of the trial (t_7_). Compliance will be calculated based on the following formula:

$$ \mathrm{Compliance}\ \mathrm{rate}:\left(\mathrm{Tablets}\ \mathrm{taken}/\mathrm{Tablets}\ \mathrm{prescribed}\right)\times 100 $$

The compliance less than 80% will be classified as poor compliance [37].

### The minimal loss to follow-up

We will use the following measures to ensure minimal loss to follow-up:

- We will include only patients willing to remain in Ahvaz for at least 6 weeks after randomization.
- We will exclude patients who cannot commit to the study for at least 6 weeks after randomization.
- We will also follow all patients by weekly phone calls and intermediate visits (t_3_).
- Patients will be instructed and asked to complete a daily calendar immediately after the consumption of their assigned treatments.

### Statistical analysis

The SPSS software (Version 16, SPSS Inc., and Chicago, IL, USA) will be used for statistical analysis. Data will be reported as the mean and standard deviation for normally distributed variables, the median and interquartile range for non-normally distributed variables, and frequencies for nominal variables. We will use the Kolmogorov-Smirnov test to determine the distribution of quantitative data. Chi-square test and Fisher’s exact test will be used to determine differences in frequencies of nominal variables. We will use the independent sample *t* test and Wilcoxon rank-sum test for within-group comparisons and paired sample *t* test and Mann-Whitney *U* test for between-group comparisons. We will also use the analysis of covariance (ANCOVA) test to adjust the potential confounding factors, such as a change in anxiety levels, physical activity, and intake of energy and nutrients, especially for FODMAPs, during the study period.

The binary logistic regression test will be used to calculate the multivariable-adjusted odds of improvement in the primary outcome with propolis supplementation. According to the patterns of missing data, a suitable multiple imputation approach will be followed for completing missing data. We will use an intention-to-treat analysis as our primary analytic approach. Linear mixed model ANOVAs may be used as a substitute statistical analysis method [38]. Statistical differences will be shown by *P* values and 95% confidence intervals. A *P* value < 0.05 will be considered statistically significant.

### Subgroup analyses

Not applicable.

### Supplementation

Each propolis tablet contains 450 mg Iranian green propolis extract, including 100 mg polyphenol compounds and 67 mg flavonoids. Propolis sample is obtained from honey bee colonies located in Rasht, the north area of Iran. The propolis and placebo will have the same shape, color, odor, taste, size, Lot number, and container and will be produced by the School of Pharmacy, Mashhad University of Medical Sciences, Iran. Also, all containers will be the same in terms of shape, color, odor, size, and weight and will be kept inside consecutively numbered, opaque, sealed envelopes.

## Discussion

Up to now, propolis has several beneficial effects on human health [39]. Many ingredients have been identified in propolis, such as flavonoids, aromatic aldehydes, lignans, esters, fatty acids, amino acids, vitamins, minerals, and phenolic acids [40]. Several studies showed the advantageous effects of propolis and its components in the treatment of GI diseases [41], such as oral mucositis [42], oral microbiota [43], ulcerative colitis [44], gastric ulcers [19], and cancers [45]. Nevertheless, little attention has been paid to IBS. Propolis is a rich source of prebiotics that can improve the intestinal barrier and subsequently can prevent pathogens, toxins, and bacterial dislocation from gut to blood [46], and also they have anti-inflammatory properties [47].

IBS has an inflammatory nature [8]. Considering its pathophysiology, we hypothesize that propolis might affect bowel habit changes in patients with IBS, including bowel movement, stool consistency, efficacious, frequency, abdominal pain, and bloating. Based on this hypothesis, we designed this study.

The novel characteristics of this clinical trial include the following: (1) This study might be a novel treatment for IBS disease for the first time in Iran and worldwide. (2) Unfortunately, IBS is prevalent in Iran, so that we predict this project might have an excellent adhesion [48].

If propolis supplement is shown effective in the management of IBS, it would be a novel therapy in individuals with IBS. Furthermore, by providing effective treatment, it could not only reduce the symptoms of IBS but also reduce the health care costs associated with repeated referral and unnecessary investigations. Because there is no effective drug for treatment modality of IBS, if propolis supplementation improves symptoms of IBS, in the future, we can design several protocols to study propolis ingredients separately, to find more effective substances. Moreover, it can be widely used due to safety and low price.

The absence of biomarkers for diagnosis, therapy evaluation, and treatment of IBS is the main limitation in the field of IBS in clinical researches. However, we will use the international *Rome IV diagnostic Questionnaire* as a validated and latest diagnosis instrument for IBS, along with *IBS quality of life Questionnaire* and *IBS Severity Index* for evaluation of IBS treatment. Another limitation of the current study is the absence of compliance biomarker for propolis intake. However, we will assess compliance with treatments by a daily calendar completed and counting the returned tablets. This clinical trial will be strengthened by stratified block randomization, double-blind and placebo-controlled design, and adjusting the results for potential confounding factors.

### Trial status

Enrollment; protocol version: 2 on 13 February 2020; the recruitment began on 31 July 2019 and will be approximately completed on 18 June 2020.

## Supplementary information

**Additional file 1.** SPIRIT 2013 Checklist.

**Additional file 2.** The consent form.

## Data Availability

The first and corresponding authors will have access to interim results and make the final decision to terminate the trial. The non-identifiable individual patients’ data will become available to other researchers in academic institutions.

## Abbreviations

ANCOVA: Analysis of covariance
BAI: Beck Anxiety Inventory
BMI: Body mass index
BSFS: Bristol stool form scale
CD: Celiac disease
FODMAPs: Fermentable oligosaccharides, disaccharides, monosaccharides, and polyols
GI: Gastrointestinal
IBD: Inflammatory bowel disease
IBS: Irritable bowel syndrome
IBS-C: IBS with constipation
IBS-D: IBS with diarrhea
IBS-QOL: IBS quality of life
IBSSI: IBS Severity Index
MET: Metabolic equivalent
SPIRIT: Standard Protocol Items: Recommendations for Interventional Trials
WC: Waist circumference
