# Supplementary material for: Does propolis affect the quality of life and complications in subjects with irritable bowel syndrome (diagnosed with Rome IV criteria)? A study protocol of the randomized, double-blinded, placebo-controlled clinical trial
Source: Trials. 2020 Aug 5;21:698. doi: 10.1186/s13063-020-04615-5 (PMC7405434; doi:10.1186/s13063-020-04615-5)
Supplement: Supplementary file 2 — Additional file 2. The consent form. [file 13063_2020_4615_MOESM2_ESM.docx]

***Patients Consent Form***

Date: ………

| ***Study title:*** Does propolis affect the quality of life and complications in subjects with irritable bowel syndrome (diagnosed with Rome IV criteria)? A study protocol of the randomized, double-blinded, placebo-controlled clinical trial |
| --- |
| ***Research Type:*** Double-blind randomized clinical trial. |
| Dear Sir/ Madam,  You are invited to participate in a research study conducted by the Tabriz University of Medical Sciences under the supervision of the Ethics Committee of the Tabriz University of medical sciences and funded by the Research affaire of the Tabriz University. Before you decide whether or not to participate, the research briefly explains why this research is being conducted and what this research entails.  Please read carefully the following research information. If you need much more explanation please ask us, and don't hurry to decide whether to participate in this research or not. |
| 1. **What is the purpose of this study? How will it be done? (Purpose and Method)**   The aim of this study is to investigate the effect of propolis supplementation on dietary intakes, quality of life, and severity of disease in patients with irritable bowel syndrome (IBS). At the beginning, all participants will be taught how to use supplements and fill out questionnaires, and anthropometric assessments including height, weight, and waist circumference will be measured. Before and after intervention, patients’ quality of life, severity of disease, physical activity, and anxiety state will be measured by questionnaires. Each patient will be fill out a three-day food record at baseline, in the middle, and at the end of the study. Patients will be requested to record all foods, and drinks consumed, with their amount for 3 consecutive days, including one weekend. Questionnaires will assess the effect of supplementation on severity of disease, quality of life, and dietary intakes pre- and post-supplementation. If you would like to know the outcomes of the study it will be provided to you at the end of the research. The supplements will be provided to you freely. |
| 1. **Why am I selected?**   You was selected based on you were recognized eligible according to the inclusion criteria of this research. |
| 1. **What is the benefits of this research for participants?**   Recent studies have shown that propolis supplement has the antioxidant and probiotic properties that have beneficial effects on the gut intestinal health. |
| 1. **Does the use of propolis have any side effects?** So far, no clinical studies have shown any side effects of propolis in this administration dose. |
| 1. **Will my information in this study be confidential?**   Your participation in this study and the information/data you provide to us will remain strictly confidential. Throughout the study, an identification number or code will be assigned to each patient and all data will remain anonymous. |
| 1. **What should I do if I want to participate in this study?**   If you agree to participate in this study, you will require to complete the informed consent form and return it to the researcher. |
| **If you agree to participate to this study, please put crosscheck:** |
| - I confirm that I have read and understand the patients consent form and I have been given the opportunity to ask any question that I have about the participation in this research. |
| - I know that my participation in this research is voluntary. I also know that I can give up research whenever I want. |
| - I agree to participate in this research. |

**Participant name and last name: ……………………. Signature: …………….**

**Reacher name and last name: ………………………. Signature: …………….**

Thank you for taking the time to read this form.
